# Tamoxifen Modulates the Immune Landscape of the Tumour Microenvironment: The Paired Siglec-5/14 Checkpoint in Anti-Tumour Immunity in an In Vitro Model of Breast Cancer

**DOI:** 10.3390/ijms24065512

**Published:** 2023-03-14

**Authors:** Przemyslaw Wielgat, Karol Rogowski, Robert Czarnomysy, Natalia Wawrusiewicz-Kurylonek, Karolina Narejko, Krzysztof Bielawski, Halina Car

**Affiliations:** 1 Department of Clinical Pharmacology, Medical University of Bialystok, Waszyngtona 15A, 15-274 Bialystok, Poland; 2 Department of Synthesis and Technology of Drugs, Medical University of Bialystok, Kilińskiego 1, 15-089 Bialystok, Poland; 3 Department of Clinical Genetics, Medical University of Bialystok, Waszyngtona 13, 15-089 Bialystok, Poland; 4 Department of Experimental Pharmacology, Medical University of Bialystok, Szpitalna 37, 15-295 Bialystok, Poland

**Keywords:** Siglec, breast cancer, microenvironment, tamoxifen, monocytes

## Abstract

Since the role of sialome–Siglec axis has been described as a regulatory checkpoint of immune homeostasis, the promotion of stimulatory or inhibitory Siglec-related mechanisms is crucial in cancer progression and therapy. Here, we investigated the effect of tamoxifen on the sialic acid–Siglec interplay and its significance in immune conversion in breast cancer. To mimic the tumour microenvironment, we used oestrogen-dependent or oestrogen-independent breast cancer cells/THP-1 monocytes transwell co-cultures exposed to tamoxifen and/or β-estradiol. We found changes in the cytokine profiles accompanied by immune phenotype switching, as measured by the expression of arginase-1. The immunomodulatory effects of tamoxifen in THP-1 cells occurred with the altered *SIGLEC5* and *SIGLEC14* genes and the expression of their products, as confirmed by RT-PCR and flow cytometry. Additionally, exposure to tamoxifen increased the binding of Siglec-5 and Siglec-14 fusion proteins to breast cancer cells; however, these effects appeared to be unassociated with oestrogen dependency. Our results suggest that tamoxifen-induced alterations in the immune activity of breast cancer reflect a crosstalk between the Siglec-expressing cells and the tumour’s sialome. Given the distribution of Siglec-5/14, the expression profile of inhibitory and activatory Siglecs in breast cancer patients may be useful in the verification of therapeutic strategies and predicting the tumour’s behaviour and the patient’s overall survival.

## 1. Introduction

According to the International Agency for Research on Cancer (IARC), breast cancer accounted for 25% of all cancers diagnosed in women worldwide in 2020 [1]. Genome-wide studies have detected several genetic variants, including *BRCA1*, *BRCA2*, *ATM*, *PALB2*, *PTEN*, *TP53*, *CDH1*, *CHEK2* and *STK11*, which are linked to an increased risk of different breast cancer subtypes [2,3]. At the protein level, the expression of sex hormone receptors, oestrogen receptors (ER), progesterone receptors (PR) and human epidermal growth factor receptor 2 (HER2) is a diagnostic and prognostic factor in the anatomic staging system and therapeutic strategy of breast cancer [4]. As well as the tumour-specific mechanisms involved in the promotion of uncontrolled growth, the interplay between malignant cells and the tumour microenvironment (TME) determines the progression of cancer and shapes the therapeutic response and resistance [5].

The Cancer Genome Atlas databases describe multiple cancer subtypes characterised by the highly heterogenous immune scenery [6]. The immune landscape of breast cancer is composed of regulatory T cells (Tregs), tumour-associated macrophages (TAMs), myeloid-derived suppressor cells (MDSC) and tumour-associated neutrophils that are recruited into the TME by tumour-derived factors. The infiltration of suppressive immune cells has been described as an indicator of invasive activity and as prognostic factors of therapeutic efficacy and overall survival. In many tumours, the molecular pathways associated with molecular mimicry recruit checkpoint molecules that mediate immune tolerance and promote the progression of cancer [7]. In breast cancer patients, several immune checkpoints, including programmed cell death protein 1 (PD-1) and cytotoxic T-lymphocyte-associated protein 4 (CTLA-4), seem to be related to the specific tumour subtype, but the relationship between expression and prognosis remains unclear [8,9,10].

The attachment of sialic acid is the final stage of glycosylation that modulates the structure and activity of the cell membrane’s glycoproteins and glycolipids. The terminal position of sialic acid in the sugar chain and its chemical and physical properties determine the role of sialoglycans in the cellular interactions underlying biological recognition processes [11,12,13,14]. The unique sialylation pattern is a control system of immune homeostasis and regulates the fundamental cellular processes, including the cell cycle, differentiation and migration [15]. In the field of immunoglycobiology, the interplay between sialic acids and Siglecs is under investigation as a regulatory mechanism of cellular recognition, and “On” and “Off” signalling in the immune system. The sialic acid immunoglobulin-like binding lectins (Siglecs) are candidate receptors for the modulation of the immune response in cancer [16]. The interaction between the tumour-specific sialoglycans and the cell-type distributed Siglecs forms a communication system that triggers suppressive signalling of the immune cells [17]. In many cancers, called immunologically “cold” tumours, the suppression of the microenvironment can be shaped by CD-33-related Siglecs that generate signals via the immunoreceptor tyrosine-based inhibitory motif (ITIM)-coupled with tyrosine–protein phosphatase non-receptor type 6 (SHP-1) and tyrosine-protein phosphatase non-receptor Type 11 (SHP-2) molecules [16,17]. Siglec-7 and Siglec-9 decrease anti-tumour immunity by interacting with highly sialylated mucins in breast cancer patients, and correlate with poor prognosis [10,18]. Therefore, the blockade of the interplay between Siglec-9 and mucin-1 (MUC1) is a potential targeting therapy aimed at the blockade of suppressive signalling in breast malignancies [19,20]. In contrast to this suppressive function, several Siglec receptor family members contain the immunoreceptor tyrosine-based activatory motif (ITAM) and transduce proinflammatory signalling via a DAP12-dependent pathway; however, their role in cancer remains not fully understood [21]. Human monocytes present the expression of paired Siglec-5 and Siglec-14 receptors characterised by the structural similarity of the extracellular domain and binding of the same ligand, whereas their functional differences are related to transduction of the opposite signal through ITIM and ITAM, respectively [22]. The gene-dependent distribution of the counterparts of paired Siglecs in the human population determines the predominance of suppressive or stimulatory mechanisms in immune regulation in individuals with invasive diseases [23]. This is of particular importance, since the sialylation pattern is known to be modulated by chemical and biological stimuli. Although the advances in immunotherapy and targeted therapy of breast cancer are in the focus of clinicians, selective oestrogen receptor modulators (SERMs) are the strategy of choice for the management of breast tumours in clinical practice [24,25]. Tamoxifen (TMX) is a non-steroid anti-oestrogen drug widely used in the therapy for and prevention of breast cancer in pre- and post-menopausal women [26]. The apoptotic effects of TMX in oestrogen receptors expressing malignant breast cells (ER+) are controlled by the antagonistic occupation of oestrogen receptors (ER) and inhibition of the cytoplasmic and nuclear oestrogen pathways underlying cell growth, survival and invasion [27]. Alongside the ER-related mechanisms, the influence of TMX on reactive oxygen species (ROS) and energy metabolism and storage (ATP) is under investigation [26,28]. Interestingly, the apoptotic effect of TMX has been observed in ER-negative (ER-) breast cancer cells as a result of the downregulation of the CIP2A/PP2A/p-Akt signalling pathway [29]. In the field of immunology, a growing body of evidence has suggested the immunomodulatory features of TMX. As shown, anti-oestrogen therapy with SERMs reverses the suppressive effects of oestrogens on immunity, which result in a decreased level of Tregs and M2 macrophages, and an enhanced level of CD4+ and CD8+ effector T cells [30,31]. The clinical importance of TMX in anti-cancer immunity has been confirmed in both ER(+) and ER(-) cancers, but the molecular mechanisms of these effects are not fully understood. Given the modulatory effects of oestrogens and anti-oestrogens on sialylation patterns and the importance of sialoglycans in breast cancer’s behaviour, the stimulatory and inhibitory Siglecs can be crucial players in immune surveillance in breast cancer patients [32,33]. On the basis of this hypothesis, we investigated the effects of TMX on the expression of paired Siglec-5 and Siglec-14 (Siglec-5/14) and their engagement in immune regulation in breast malignancies in vitro. We established a transwell co-culture model of human monocytic THP-1 cells growing with human breast cancer cells chosen according to the major features of human breast cancer and its progression. Hence, MCF-7 cells were used as non-invasive ER(+) breast malignancies, whereas MDA-MB-231 cells simulated highly invasive triple negative (ER(-), PR(-), HER2(-)) tumours. Tamoxifen is a competitive antagonist of ER and competes with 17β-estradiol at the receptor site; therefore, combined stimulation with E2 was performed to highlight and compare the opposite effects of ER ligands on immunity. Alterations in the immune phenotype of immune cells and the expression of paired Siglecs provided evidence of a regulatory mechanism dependent on sialic acid and Siglec for TMX’s activity in human breast cancer.

## 2. Results

### 2.1. The Immune Response of THP-1 Cells Exposed to TMX and E2 Treatment

**Figure 1:**
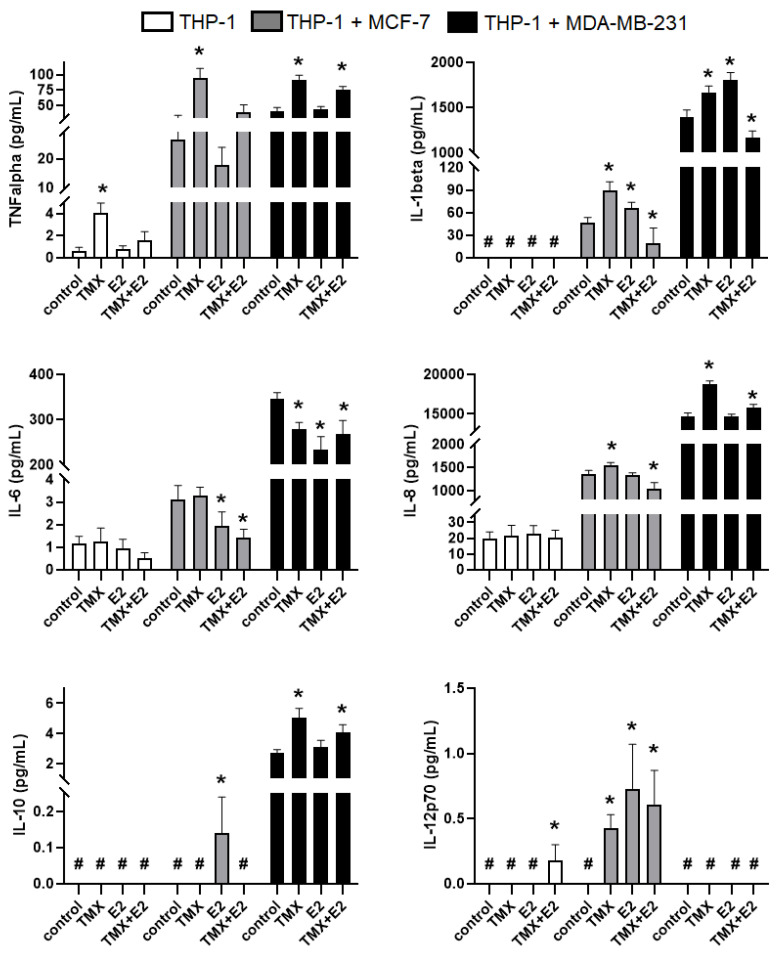
Flow cytometric analysis of monocytic THP-1 cells for the expression of TNFα, IL-1β, IL-6, IL-8, IL-10 and IL-12p70. Cells were grown in a monoculture or the presence of MCF-7 and MDA-MB-231 cells in a co-culture transwell system, and exposed to TMX (10 μM) and/or E2 (100 nM) for 24 h. Each column presents the mean concentration from 3 independent experiments. * *p* < 0.05 vs. the corresponding control group; # concentration < 0.01 pg/mL.

The immunomodulatory effect of TMX on the crosstalk between THP-1 monocytes and breast cancer cells was estimated by cytometric analysis of the expression of cytokines in cultured and co-cultured cells. The specific cytokine markers were used to evaluate the modulatory effects of TMX, E2 or TMX/E2 stimulation on the immune status of the tested culture systems. Since specific cytokines are known to be biomarkers of the M1 and M2 phenotypes, we analysed both proinflammatory and anti-inflammatory cytokine levels. Therefore, TNF-α, IL-1β, IL-6 and IL-8 were assessed as secretory biomarkers of the M1 phenotype. IL-10 was studied as the marker of the M2 phenotype. The comparison of the analysed cytokine levels, described as the amount of cytokine proteins per millilitre of cellular supernatant, revealed significant differences between the culture systems. In relation to the cytokines present in the samples of monocultured monocytes, in general, low levels of TNF-α, IL-6 and IL-8 were found, when compared with co-cultured cells, whereas IL-1β, IL-10 and IL-12p70 remained undetectable. In cell co-cultures, high levels of the tested cytokines in the experimental groups were observed, except IL-10 in the THP-1/MCF-7 and IL-12p70 in the THP-1/MDA-MB-231 cell culture subsets. Both monocultured and co-cultured THP-1 monocytes showed significantly enhanced levels of TNFα (THP-1: 4.06 ± 0.68 vs. 0.62 ± 0.32 for naïve cells; THP-1/MCF-7: 95.21 ± 14.8 vs. 26.7 ± 7.5 for naïve cells; THP-1/MDA-MB-231: 92.02 ± 7.3 vs. 41.04 ± 5.9). Similarly, the expression of pro-inflammatory IL-1β was significantly higher in TMX-treated co-cultures when compared with untreated cells (THP-1/MCF-7: 89.53 ± 11.5 vs. 11.5 for naïve cells; THP-1/MDA-MB-231: 1669 ± 68.9 vs. 1399 ± 74.10 for naïve cells). Interestingly, the expression of TNFα was not significantly changed in response to E2 in all experimental groups; however, this effect was reversed by TMX. In relation to IL-1β, the opposite effects of E2 were observed in oestrogen-dependent MCF-7-based co-cultures and oestrogen-independent MDA-MB-231-based cultures. Regarding IL-8, a significant enhancement of its expression was observed in both MCF-7 and MDA-MB-231 co-cultures exposed to TMX, and a minor effect was seen in response to E2. In response to TMX, the IL-6 in both cultured THP-1 cells and the THP-1/MCF-7 co-culture system presented similar expression levels to naïve monocytes and a significant decrease in THP-1/MDA-MB-231 co-cultures. Contrary to TNFα, IL-1β and IL-8, the expression of IL-6 was significantly reduced in response to E2 in all culture systems, which was potentiated by additional TMX in the THP-1 and THP-1/MCF-7 cultures but not in MDA-MB-231. Surprisingly, the evaluation of the expression of IL-10 in the THP-1 culture and the THP-1/MCF-7 co-culture failed due to its undetectable concentration in the analysed samples. In contrast, a significantly increased level of IL-10 was observed in TMX-treated THP-1/MDA-MB-231 co-cultures. A similar effect was observed in THP-1/MDA-MB-231 co-cultures regarding the expression of IL-12p70 (Figure 1). 

**Figure 2:**
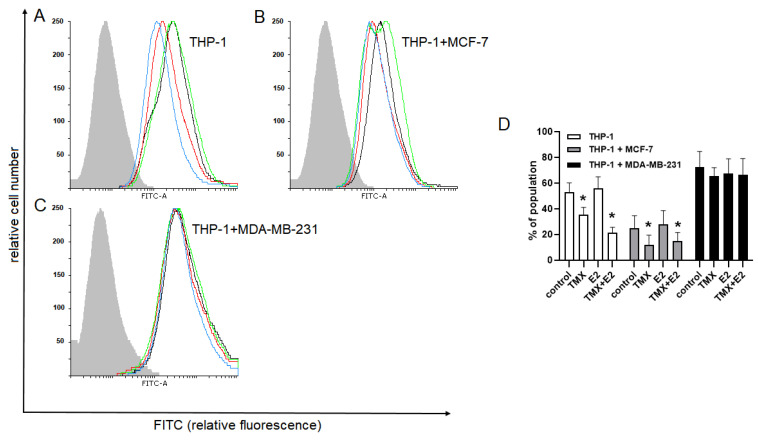
Expression of Arg-1 in monocytic THP-1 cells grown in a monoculture (**A**) or the presence of MCF-7 (**B**) and MDA-MB-231 (**C**) cells in a co-culture transwell system and exposed to TMX and/or E2. Representative histograms (**A**–**C**) and corresponding bar graphs (**D**) were derived from flow cytometric analyses of 10,000 cells and show the isotype of the control (light grey histogram), the control cells (black line), TMX-treated cells (red line), E2-treated cells (green line) and cells concomitantly treated with TMX/E2 (blue line). Data are presented as the median percentage of each population (mean of 5 separate determinations). * *p* < 0.05 vs. the corresponding control group.

Given the ability of monocytes to convert into macrophages and their engagement in the breast tumour microenvironment, we evaluated the expression of arginase-1 (Arg-1) as a marker of the M2 immunosuppressive phenotype. In the THP-1 monoculture and the THP-1/MCF-7 co-culture, the expression of Arg-1 was significantly decreased in response to TMX (THP-1: 35.4 ± 5.7 vs. 53.00 ± 7.2 for the naïve control; THP-1/MCF-7: 12.1 ± 7.4 vs. 25.1 ± 9.5 for the naïve control), whereas exposure to 100 nM E2 resulted in a non-significant increase in the expression of Arg-1 (THP-1: 56.1 ± 8.8 vs. 53.00 ± 7.2 for the naïve control; THP-1/MCF-7: 27.8 ± 10.8 vs. 25.1 ± 9.5 for the naïve control). The effects of E2 were significantly diminished when TMX was added. Surprisingly, the expression of Arg-1 in the THP-1/MDA-MB-231 co-culture was reduced in all experimental groups; however, these changes were not significant (Figure 2).

### 2.2. Effect of TMX and E2 on the Expression of Paired Siglec-5/Siglec-14 in Human Monocytic THP-1 Cells

**Figure 3:**
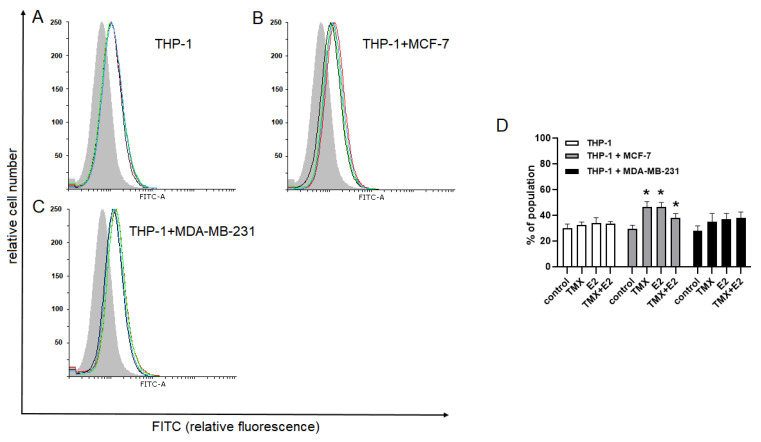
Expression of paired Siglec-5/Siglec-14 in human monocytic THP-1 cells grown in a monoculture (**A**) or the presence of MCF-7 (**B**) and MDA-MB-231 (**C**) cells in a co-culture transwell system and exposed to TMX and/or E2. Representative histograms (**A**–**C**) and corresponding bar graphs (**D**)were derived from flow cytometric analyses of 10,000 cells and show the isotype of the control (light grey histogram), the control cells (black line), TMX-treated cells (red line), E2-treated cells (green line) and cells concomitantly treated with TMX/E2 (blue line). Data are presented as the median percentage of each population (mean of 5 separate determinations). * *p* < 0.05 vs. the corresponding control group.

To evaluate the effect of TMX on paired Siglec-5 and Siglec-14, flow cytometry with a monoclonal antibody recognising the extracellular domain of Siglec-5 and Siglec-14 was used. The expression of Siglec-5 and/or Siglec-14 proteins in the THP-1 control cells was similar to those observed in naïve cells growing in the presence of MDA-MB-231 cells and was not significantly altered after exposure to TMX and E2. In the THP-1/MCF-7 co-cultures, the expression of Siglec-5/14 was significantly elevated in response to 10 μM TMX (46.50 ± 4.13 vs. 29.4 ± 2.88 for naïve cells). Interestingly, a similar effect was also observed in E2-treated cells (46.30 ± 3.59 vs. 29.4 ± 2.88), but not in combination with TMX (Figure 3).

### 2.3. Assessment of the Gene Expression of Paired Siglec-5/14 and the Related Signalling Molecules in Monocytic THP-1 Cells Exposed to TMX and E2

**Figure 4:**
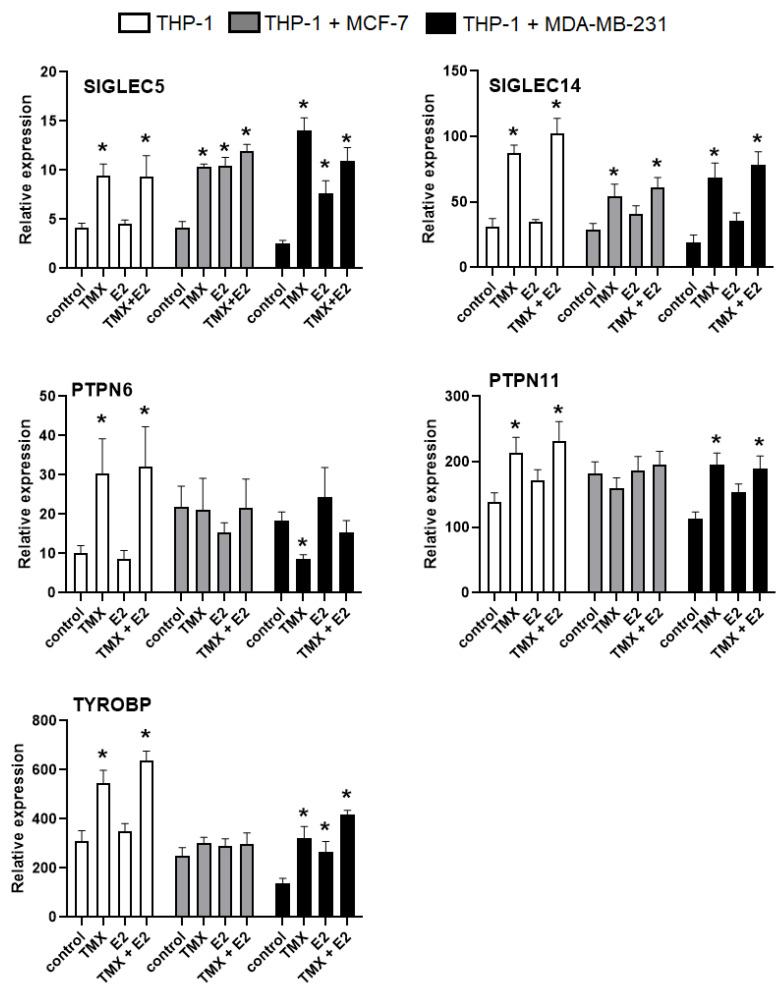
Expression of *SIGLEC5*, *SIGLEC14*, *PTPN6*, *PTPN11* and *TYROBP* mRNA in monocytes. The analysed transcripts were detected by real-time PCR in monocytic THP-1 cells grown in a monoculture and a co-culture. The housekeeping gene *GAPDH* was used as an internal loading control. Representative data show a mean of three samples. * *p* < 0.05 vs. the corresponding control group.

Given the immunomodulatory potential of SERMs and the engagement of Siglecs in breast cancer’s biology, we assessed the transcriptional regulation of the Siglec-dependent immune response induced by TMX. The analysis of mRNA transcripts for *SIGLEC5* and *SIGLEC14* confirmed their similar expression in both naïve THP-1 monocultures and co-cultures, whereas significantly increased levels were detected in TMX-treated cells. The exposure to E2 did not alter the transcripts of *SIGLEC5* and *SIGLEC14* in monocultured THP-1; however, the opposite effects were observed in co-culture systems. The expression levels of mRNA *SIGLEC14* transcripts remained unchanged in response to E2, but *SIGLEC5* levels were significantly increased in both oestrogen-dependent and -independent co-cultures. The combined treatment with TMX and E2 significantly increased the mRNA transcripts for *SIGLEC5* and *SIGLEC14* in all cell culture subsets. To evaluate the functional role of alterations in the expression of *SIGLEC5* and *SIGLEC14* genes, the genes encoding Siglec-mediated signalling pathway molecules were evaluated in this study. Among the inhibitory signalling pathway molecules, the mRNA transcripts for *SHP1 (PTPN6)* and *SHP2 (PTPN11)* were evaluated in this study. The significantly enhanced expression of both *PTPN6* and *PTPN11* was found in monocultured THP-1 monocytes exposed to TMX under a single or combined treatment, whereas significant changes were not observed in E2-treated cells. The expression of *PTPN6* and *PTPN11* transcripts in THP-1 monocytes growing in the presence of MCF-7 cells remained unchanged after exposure to TMX and/or E2. In contrast, the level of *PTPN11* transcripts in THP-1/MDA-MB-231 co-cultures was significantly higher compared with naïve cells, whereas the expression of *PTPN6* tended to be reduced. Finally, analysis of the expression of the gene encoding activatory DAP12 protein revealed a significant increase in mRNA transcripts for *TYROBP* in both the THP-1 monoculture and the THP-1/MDA-MB-231 co-culture treated with TMX. Surprisingly, both TMX and E2 did not produce changes in the *TYROBP* transcripts in the THP-1/MCF-7 co-culture (Figure 4).

### 2.4. The Recombinant Siglec-5/Fc and Siglec-14/Fc Chimeras Cover Human MCF-7 and MDA-MB-231 Cells in Response to TMX Treatment

**Figure 5:**
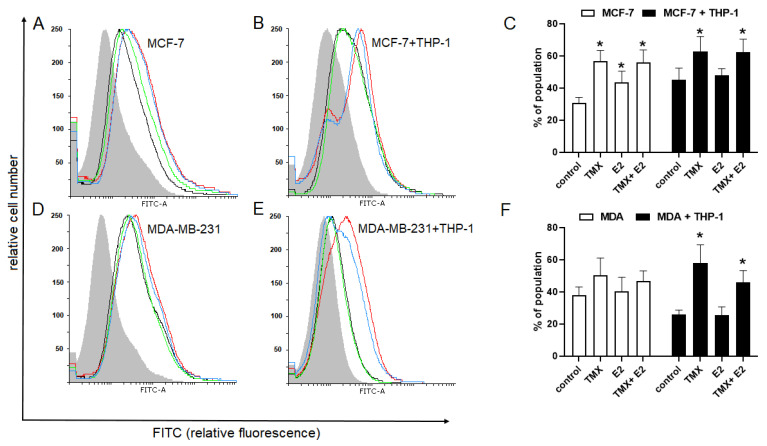
Effect of TMX and/or E2 on the binding of Siglec-5/Fc fusion protein to MCF-7 (**A**,**B**) and MDA-MB-231 (**D**,**E**) breast cancer cells grown in monocultures or co-cultures. Representative histograms and the corresponding bar graphs (**C**,**F**) show the isotype of the control (light grey filled histogram), the control cells (black line), TMX-treated cells (red line), E2-treated cells (green line) and cells concomitantly treated with TMX/E2 (blue line). Data are presented as the median percentage of each population (mean of 5 separate determinations). * *p* < 0.05 vs. the corresponding control group.

According to the general hypothesis, the surface sialoglycans of the tumour cell play a crucial role in immunosurveillance. Here, we analysed the effect of TMX on the binding of soluble recombinant Fc chimeric proteins of inhibitory Siglec-5 and stimulatory Siglec-14 in human breast cancer cell lines. Both MCF-7 and MDA-MB-231 cells interacted with Siglec Fc chimaeras in all the tested culture systems. Despite the structural similarity and the same ligand-binding ability, several differences between the binding of Siglec-5 and Siglec-14 Fc proteins have been found. In detail, the binding of Siglec-5 Fc fusion protein to MCF-7 cells was significantly increased in response to TMX and/or E2 exposure (MCF-7: TMX, 56.90 ± 6.55; E2, 43.4 ± 7.1; TMX/E2, 55.8 ± 7.9 vs. 30.7 ± 3.53). In MDA-MB-231 cells, similar, but not significant, alterations were observed. The binding of the Siglec-5 Fc chimaera in both the MCF-7/THP-1 and MDA-MB-231 co-cultures was significantly increased in response to TMX alone or in combination with E2 (Figure 5). 

**Figure 6:**
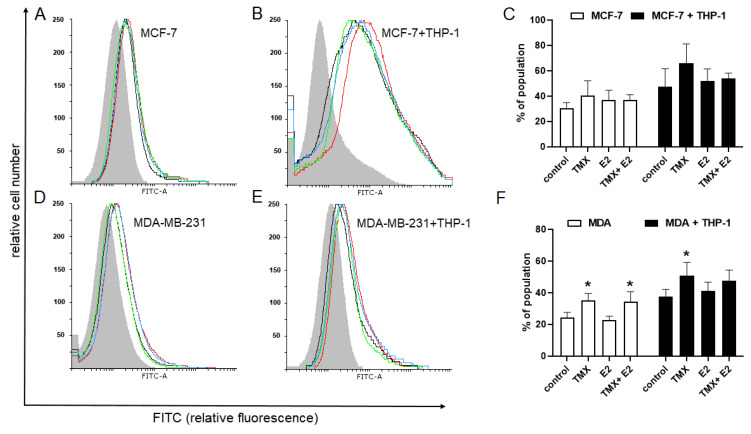
Effect of TMX and/or E2 on the binding of Siglec-14/Fc fusion protein to MCF-7 (**A**,**B**) and MDA-MB-231 (**D**,**E**) breast cancer cells grown in monocultures or co-cultures. Representative histograms and the corresponding bar graphs (**C**,**F**) show the isotype of the control (light grey filled histogram), the control cells (black line), TMX-treated cells (red line), E2-treated cells (green line) and cells concomitantly treated with TMX/E2 (blue line). Data are presented as the median percentage of each population (mean of 5 separate determinations). * *p* < 0.05 vs. the corresponding control group.

The Siglec-14 Fc fusion protein, contrary to Siglec-5 Fc chimera, showed significantly increased affinity to MDA-MB-231 but not MCF-7 cells exposed to TMX alone or the combined TMX/E2 treatment. In relation to the co-culture systems, TMX caused a significant increase in the binding of the Siglec-14 Fc chimera in oestrogen-independent MDA-MB-231 cells, but not in the MCF-7/THP-1 system (Figure 6).

## 3. Discussion

Despite the progress in cancer immunotherapy, the SERMs are a part of conventional therapeutic strategy for oestrogen-dependent breast tumours [34,35]. The interference with oestrogens and the inhibition of oestrogen-related signalling pathways emphasise the anti-proliferative activity as the main on-target effect of TMX on malignant breast cells [36]. Beyond the expected tissue-specific therapeutic results, several ER-unrelated effects as toxic responses to TMX have been observed. This is of particular importance in the context of the usage of SERMs in the management of ER(-) breast tumours [37]. The role of immune cells in the progress of pathology is closely related to alterations in the functional phenotype featured by two opposite activation states. Multiple physio-pathological signals, including the presence of malignant cells and pharmacological stimulation, promote the polarisation of the immune cells towards M1 or M2 phenotype [38]. Whereas the M1 phenotype presents with proinflammatory and anti-tumour activity, the M2 phenotype involves the tumour-promoting processes, including immunosuppression and tissue remodelling [39,40]. Among the off-target effects, the broad immunomodulatory influence of TMX in immune cells, including the macrophages, was associated with promotion of the inflammatory phenotype without an influence on cell viability [40]. In this study, we focused on the potential engagement of Siglecs in the regulatory mechanisms of TMX-induced immunomodulation. Given the on- and off-target action of TMX, we developed a transwell co-culture system based on breast cancer cells classified according to their ER expression and dependency on E2; these were cultivated in the presence of human monocytes. In the field of clinical TMX administration, on- and off-target effects are closely related to the concentration and timing of the therapy. The on-target effects in ER(+) breast malignancies, including cell toxicity, were observed during long-term treatment with TMX at a daily dose of 20–40 mg, and a final serum concentration of 0.2–0.5 μM and 3–5 μM in serum and tissue, respectively. Nevertheless, short-term administration of TMX at a daily dose of 250–500 mg, which corresponds to a concentration of 10–20 μM in tumour tissues, has multiple off-target effects on ER(-) cancers [40]. Hence, we referenced the effects of 10 μM TMX observed in this study to the on- and off-target effects in vivo. Our analysis of the monocytic immune activity demonstrated a modulatory effect of TMX on the production and secretion of pro- and anti-inflammatory cytokines. The quantitative examination of the cytokine profile confirmed the pro-inflammatory potential of TMX, as shown by the enhanced expression of TNFα, IL-1β and IL-8. Moreover, THP-1 monocytes showed cytokine expression levels that were several times higher in the presence of both E2-dependent and E2-independent cells than in monocytes cultured alone. Several studies showed that the immune consequences of TMX exposure in macrophages are expressed as the potentiation of the inflammatory response, autophagy and phagocytosis regulated by off-target mechanisms. The study by Pepe et al. demonstrated that TMX promotes the maturation of IL-1β and its secretion by the macrophages as a result of its direct influence on the formation of caspase-1 [41]. According to Sfogliarini et al., the phosphatidylinositol 3-kinase/nuclear factor erythroid 2-related factor 2 (PI3K-NRF2) pathway and multiple molecular mediators, including protein kinase C (PKC) and oxidative stress molecules, may represent a host-mediated mechanism that leads to the beneficial TMX-induced immune activity against pathogens [40]. These effects are related to the functionally activated phenotypes of monocyte-derived macrophages quantified by an analysis of their M1/M2 activation markers. Since a reduction in intracellular arginine is known to dampen inflammation and phagocytosis, the high expression of arginase-1 is one of the primary characteristics of M2 polarisation in cancer immunosurveillance [42]. In this study, we defined the immune status of THP-1 cells according to the M1/M2 polarisation state by an assessment of the intracellular expression of Arg-1. In response to 10 μM TMX, the expression of Arg-1 significantly decreased in monocultured monocytes and showed an increasing tendency in the group treated with 100 μM E2.

Interestingly, the evaluation of the expression of Arg-1 revealed the opposite responses of THP-1 monocytes to external stimuli within the tested co-cultures. The study by Dahmani et al. revealed that the phenotypic functional activities of monocytes were modified after co-culturing with primary human breast cancer cells [43]. In the presence of MCF-7 but not MDA-MB-231 cells, THP-1 cells showed a strong decrease in the expression of Arg-1 when compared with monocytes cultured alone. A comparison of the expression levels of Arg-1 in all tested experimental groups with the THP-1/MCF-7 culture and the high level of pro-inflammatory IL-8, IL-1β and TNFα confirmed the promotion of the M1 phenotype in response to exposure to TMX. On the contrary, the expression of Arg-1 was higher in THP-1/MDA-MB-231 co-culture in comparison with the THP-1 monoculture; however, no significant alterations within the experimental groups were found. These results suggest that the activation of infiltrated monocyte-derived macrophages in the tumour microenvironment can be dependent on both on- and off-target mechanisms. This is of particular importance, since high arginase activity has been found as a biological marker of breast cancer’s progression [44].

Multiple studies on breast cancer’s biology have focused on the alterations in the cellular composition of the microenvironment and the importance of its molecular signatures in clinical practice. Given the importance of host immunity in the progression of cancer, the transformation of an immunosuppressive tumour milieu into an immunogenic effector is a therapeutic opportunity. Hence, several studies have revealed that both inhibiting the recruitment of TAM and reprogramming TAM from the M2 to the M1 phenotype prevented tumour progression and improved the efficacy of standard chemotherapy [45]. There is increasing evidence that administration of TMX directly affects the tumour-promoting function of MDSC and dendritic cells (DCs), and increases the number of effector and cytotoxic T cells infiltrating the tumour niche [46]. This phenomenon has been observed in multiple TMX-insensitive malignant cells, including melanoma, lung and mammary cancers, and is correlated with prolonged overall survival [46,47]. Nevertheless, the fundamental mechanisms that underlie the changes within the immune cell pool produced by TMX are not fully understood. Recent advances in the immunobiology of breast cancer have revealed the recruitment of several checkpoints in the regulatory mechanism of tumour-infiltrating Tregs. As shown, upregulation of PD-L1-specific T cells is essential for maintaining the inflammatory response through the suppression of Tregs’ functions [48]. It has been reported that some conventional chemotherapeutics could change the expression of checkpoints; however, little is known about the clinical implications of this phenomenon during systemic therapy in breast cancer. Several data have confirmed the TMX-induced disturbances in the expression and function of PD-1 and CTLA-4. Observations by Huhn et al. showed that oestrogen deprivation enhanced the expression of PD-L1, accompanied by alterations in the transcription of inflammatory cytokines in ER+ breast cancer [49].

Since the expression of Siglec family members has been found in the major immune cells, their role in the maintenance and pathology of immune homeostasis has been extensively studied. The diversity in the cell-specific distribution of Siglecs and their affinity to the tissue-specific ligands underlie the function of immune cells in self and non-self biological recognition and balanced immune control [14]. The interaction between Siglec-expressing cells and aberrantly sialylated components of the tumour microenvironment recruits the intracellular signal transduction systems that form a mechanism of immune surveillance in tumours. The engagement of inhibitory Siglecs negatively modulates the immune responses by promoting the formation of an immunosuppressive microenvironment and the tumour’s immune escape [17]. Thus, the aim of combined cancer management can be modulation of the tumour microenvironment for the reversion of suppressed cancer immunity, in addition to anti-proliferative effects of conventional chemotherapeutics [50].

Given the widespread application of SERMs in the control and management of breast malignancy, we studied the importance of Siglecs in TMX-modulated immunity. Our previous studies confirmed the modulatory effects of chemotherapeutics on the expression and function of the Siglecs widely expressed in immune cells, including human monocytes, which are recruited into the pathological process. Several data confirmed the modulatory effects of conventional therapeutics the expression of paired Siglecs, including Siglec-5/14 and Siglec-11/16. However, the Siglec family receptors have not been extensively studied in the context of the immunomodulatory functions of TMX. This study was the first investigation of the expression of paired Siglec-5/14 in TMX-modulated immunity [51,52,53]. Here, we examined the expression of genes encoding paired Siglec-5/14 and the associated signalling molecules in human monocytes exposed to TMX in the presence of malignant cells. Our previous study confirmed the expression of transcripts for *SIGLEC5* and *SIGLEC14* in THP-1 monocytes [51]. In this study, quantitative real-time PCR showed the modulatory influence of TMX on *SIGLEC* transcripts in the analysed cell populations. Despite the effect of TMX on *SIGLEC* transcripts being found in both monocultured and co-cultured monocytes, the *SIGLEC14* transcripts showed the highest relative expression levels in all analysed groups. The expression of Siglec-5 and Siglec-14 proteins was evaluated by flow cytometry after cell labelling with a specific monoclonal antibody recognising the extracellular domains of both membrane receptors. Indeed, significant alterations in the membrane expression of Siglec-5/14 were found in THP-1 cells grown with oestrogen-dependent MCF-7 cells. This phenomenon can be explained by the high specificity of the antibody used and its affinity toward Siglec-5 but its partial cross-reactivity to Siglec-14. Recent findings in the field of the immunobiology of breast cancer have shown that the predominant engagement of Siglecs and the broad expression of sialoglycans are closely associated with malignant transformation and are a part of the immune evasion strategy [54,55,56]. Since the expression of highly sialylated mucins are markers of malignant cells’ secretory activity, their interplay with Siglec-9 is considered the main regulator of immune tolerance in breast cancer patients [20]. Likewise, the analysis of breast tumour-infiltrating cell populations showed that highly aggressive breast cancers, including triple negative tumours, are characterised by the presence of Siglec-7- and Siglec-10-positive cells in the tumour stroma [57]. This was correlated with the high content of sialoglycoproteins in the fibrotic areas within the tumour tissue. The investigation by Shaffi revealed that breast tumours express approximately 70% of Siglec-15-positive cells in the tumour stroma, which was found to be mutually exclusive to PD-L1 [56]. This may suggest the possible therapeutic benefits of Siglec-15 targeting in patients with a negative response to PD-1/PD-L1 blockade. Furthermore, functional studies on the importance of Siglecs in the progression of cancer have indicated the predominant role of immunosuppressive Siglec receptors and their cognate ligands. Beatson showed that the infiltration of Siglec-9-expressing cells into MUC-1 containing breast tumours triggered an immunosuppressive effect through the recruitment of SHP-1 and SHP-2 signalling molecules [58]. However, the aberrant sialylation in MUC-1 resulted in the synthesis of membrane-bound glycoforms of MUC-1 decorated by multiple short, sialylated O-linked glycans (MUC-1-ST). As shown, the binding of MUC-1-ST to inhibitory Siglec-9 has the opposite effects to those induced by MUC-1. The interplay between MUC-1-ST did not activate the SHP-1/SHP-2 signalling pathway, but calcium flux-associated cell activation was observed [59]. Although our study did not investigate the role of Siglecs in direct co-culture systems, the results obtained here seem to confirm this phenomenon. The results suggest that the TMX/E2-induced alterations in the structure and expression of secreted soluble sialoglycans can lead to the modulation of the immune activity of co-cultured monocytes, as shown by differences in the expression of genes encoding Siglecs-coupled signalling molecules. As we have shown above, the high relative expression of the *SIGLEC14* gene transcripts, in contrast to Siglec-5, was accompanied by the high relative expression of *DAP12* gene transcripts. The variable expression of paired Siglec counterparts in the human population reflect the mechanism of genetically determined immune responses. As shown, the patients with wild-type alleles and predominant expression of the *SIGLEC14* gene develop an acute immune response in invasive and inflammatory disorders, whereas the lack of the Siglec-14 receptor and the overexpression of Siglec-5 promoted immunosuppression and correlated to low production and secretion of cytokines [59]. Our results seem to support the hypothesis that the conventional drugs used in standard cancer therapy can modulate the glycosylation mechanisms and exert different effect on immune-controlling systems [60]. The various effects on the immune function of THP-1 monocytic cells, accompanied by the altered expression of Siglecs in the gene and protein levels, reflect the suggested immunomodulatory properties of TMX. Interestingly, the TMX-induced alterations in the binding of Siglec Fc chimeras suggested the structural changes in the cell membrane’s sialome in an oestrogen-dependent manner.

In the field of the role of the Siglec checkpoint in anti-tumour immunity, there are several limitations in the present study. Given the complexity of the mechanisms underlying the formation and progression of tumour, cell lines are crude models of tumours and cannot reflect all the features and heterogeneity of tumour. The molecular features of the cell lines used, including the expression of sex hormone receptors and the expression of the wild-type *BRCA1* gene, remained the same in tissue tumours and allowed us to perform a preliminary comparative analysis based on the oestrogen-dependent and oestrogen-independent mechanisms. The development of breast cancer animal models seems to reflect the complex mechanism underlying cellular interactions within the tumour tissues and would complement in vitro experiments. However, the strong differences in the structure and distribution of Siglecs in animal and human cells, as well as the lack of paired receptors in mice and rats, limit the usefulness of animal models in this field. Siddiqui suggested that novel functions of Siglecs, including sialic acid-independent interactions and non-classical signalling, and the expression and function of non-immune cells open a new experimental field for studies on their potential application in therapy [61].

## 4. Materials and Methods

### 4.1. Cell Cultures and Treatment

Human breast cancer cells (MCF-7 and MDA-MB-231) were obtained from ATCC and were cultured in Roswell Park Memorial Institute medium (RPMI-1640, ATCC, Manassas, VA, USA) supplemented with 10% foetal bovine serum and 1% penicillin/streptomycin stock. Human monocytic THP-1 cells were cultivated in an RPMI-1640 medium with the addition of 10% heat-inactivated foetal bovine serum, a 1% penicillin/streptomycin mixture and 2-mercaptoethanol (Gibco, Thermo Fisher Scientific, Inc., Waltham, MA, USA) to a final concentration of 0.05 mM. Both breast cancer cells and human monocytes were incubated in a 5% CO_2_ atmosphere at 37 °C. For experiments performed in monocultures, cells were seeded in 6-well plates at a density of 0.3 × 10^6^/1.5 cm^2^ and cultured to reach a confluency of 80% in phenol red-free RPMI-1640 complete medium. To obtain the transwell co-culture system, THP-1 monocytic cells were cultured in 6-well plates, and MCF-7 or MDA-MB-231 were seeded in the inserts placed in the upper part of each well at a ratio of 10:1 (THP-1:MCF-7/MDA-MB-231). The cells were separated by a 0.4 μm microporous membrane to prevent physical cell–cell contact; however, chemical communication was preserved. Both monocultured and co-cultured cells were exposed to 17β-estradiol (E2) (Merck, Darmstadt, Germany) and/or TMX (Dr. Ehrenstofer; Augsburg, Germany) for 24 h. To prepare the stock solution, TMX and E2 were newly dissolved in sterile dimethyl sulfoxide (DMSO), (Merck, Darmstadt, Germany) at a concentration of 0.053 M. Then the stock solution of TMX and E2 was added to the culture medium to obtain a final concentration of 10 μM and 100 nM, respectively.

### 4.2. Immune Response and Expression of Siglecs in Monocytic THP-1 Cells

The immune status of THP-1 monocytes can be described by their secretion of cytokines and their capacity for conversion into macrophages of the M1 or M2 phenotypes. The levels of IL-1β, IL-6, IL-8, IL-10, IL-12p70 and TNFα were measured using the Cytokine Bead Array (CBA) Human Cytokine Kit (Beckton Dickinson Biosciences, San Jose, CA, USA) according to the manufacturer’s protocol. In brief, 50 μL of the sample, 50 μL of the assay beads and 50 μL of PE-labelled antibodies were incubated at room temperature for 3 h and analysed using a BD FACS Canto II flow cytometer and FCAP Array v3 software (both from BD Biosciences Systems, San Jose, CA, USA). The phenotype of the immune cells was determined by an assessment of the expression of arginase-1 (Arg-1) as a marker of the M2 phenotype. Briefly, naïve and E2/TMX-treated THP-1 cells were diluted to 10^5^ per sample, permeabilised in 0.1% Triton X-100 and incubated with Arg-1 antibody (Invitrogen, Carlsbad, CA, USA; 2.6 μg/mL) or the isotype’s antibody as a negative control for 30 min at 4 °C. Cells were stained with an appropriate secondary fluorescent antibody and analysed in a Becton Dickinson flow cytometry system. For the assay of paired Siglec-5/14 expression, THP-1 cells were incubated with a monoclonal Siglec-5/Siglec-14 antibody (Clone 1A5; MyBioSource Inc., San Diego, CA, USA; 5 μg/mL) and analysed cytometrically. The 1A5 clone was designed to detect the inhibitory counterpart of paired Siglec-5/14; however, it also recognises human Siglec-14 due to a highly similar sequence to Siglec-5 within the first two Ig-like domains.

### 4.3. Real-Time PCR

For the real-time PCR, total RNA was isolated from naïve and stimulated THP-1 cells using the RNeasy Mini Kit (Qiagen, Hilden, Germany), according to the manufacturer’s specifications. The quantity of RNA was verified by spectrophotometry, based on the ratio of absorbance at 260 nm and 280 nm. One microgram (1 μg) of total RNA was used to prepare the cDNA by the SuperScript™ First-Strand Synthesis System for RT-PCR (Invitrogen, Carlsbad, CA, USA) in the MJ Research Thermal Cycler (PTC-200, Watertown, MA, USA). The levels of the primary transcripts of human genes (*SIGLEC5*, *SIGLEC14*, *TYROBP* (formerly *DAP12*), *PTPN6* (formerly *SHP1*) and *PTPN11* (formerly *SHP2*) were estimated by real-time PCR using commercial primers from the Qiagen QuantiTech Assay, namely HS_SIGLEC5_1_SG; HS_SIGLEC14_1_SG; HS_TYROBP_1_SG; HS_PTPN6_1_SG; HS_PTPN11_1_SG and HS_GAPDH_1_SG, as these are among the most commonly used reference genes. The analysis was performed using the QuantiTech SYBR Green PCR Master Mix (Qiagen) in a CFX96 Real-Time PCR Detector (Bio-Rad, Hercules, CA, USA). The non-specific PCR products were eliminated by an analysis of the melting curve. Data were calculated using the comparative CT method for relative quantification of all gene transcripts.

### 4.4. Assay of the Binding of Siglec-5/14 to Glycans 

The TMX-induced changes in the sialylation pattern of the cell’s surface and its role in sialic acid-dependent immune regulation were investigated using a test of Siglec-5/14’s affinity to the cell membranes. Both MCF-7 and MDA-MB-231 breast tumour cells were incubated with recombinant human Siglec-5/Fc and Siglec-14/Fc chimeric proteins (both from R&D Systems; Minneapolis, MN, USA; 5 μg/mL) followed by the appropriate fluorescein (FITC)-conjugated IgG secondary antibody (Jackson ImmunoResearch; Ely, Cambridgeshire, UK; 2 μg/mL). The binding capacity of Siglec-5/14 was evaluated by flow cytometry.

### 4.5. Statistical Analysis

The obtained experimental data within monocyte populations and breast cancer cells were evaluated using one-way ANOVA with Bonferroni’s post test using Instat (GraphPad Software Inc., San Diego, CA, USA). For each group, at least three independent experiments were performed. The results are expressed as the percentage of the population ± SD. Statistical differences were deemed to be significant at *p* < 0.05.

## 5. Conclusions

The tumour microenvironment is a complex and evolving system of cellular and molecular components that control the tumour’s biology. The balance between pro- and anti-tumour activities forms the fate of the tumour. However, the time-dependent alterations in tumour cells result in immune evasion that is functionally correlated with the hiding or loss of cancer antigens and the interplay between the tumour’s ligands and the co-inhibitory immune receptors. According to the tested hypothesis, exposure to TMX plays a pivotal role in the mobilisation of immune cells in breast cancer. The stimulatory effects of TMX on the host’s immunity correspond to the altered function of Siglec receptors. The relationship between the changes in TMX-induced immune activity and the interplay between Siglecs and sialoglycans suggests the existence of sialic acid-based mechanisms that regulate the functional alterations in cancer immunosurveillance. In this perspective, the future experiments in this field would provide for the estimation and comparison of Siglecs’ expression and Siglec-related changes in both malignant and immune cells isolated from the breast cancer types characterised by different molecular and genomic profiles. The consideration of Siglec receptors in the regulation of M1/M2 phenotypes within breast tumour therapy may reduce immune evasion and improve the survival prospects. Thus, the expression profiles of inhibitory and activatory Siglecs in breast cancer patients may be useful in verification of the therapeutic strategy and predictions of the tumour’s behaviour and the patient’s overall survival.

## Data Availability

The data presented in this study are available on request from the corresponding author.
